# Current Preoperative Management of Vulvar Squamous Cell Carcinoma: An Overview

**DOI:** 10.3390/cancers16101846

**Published:** 2024-05-11

**Authors:** Luigi Della Corte, Valeria Cafasso, Maria Chiara Guarino, Giuseppe Gullo, Gaspare Cucinella, Alessandra Lopez, Simona Zaami, Gaetano Riemma, Pierluigi Giampaolino, Giuseppe Bifulco

**Affiliations:** 1 Department of Neuroscience, Reproductive Sciences and Dentistry, School of Medicine, University of Naples Federico II, 80131 Naples, Italy; luigi.dellacorte@unina.it; 2 Department of Public Health, University of Naples Federico II, 80131 Naples, Italy; va.cafasso@studenti.unina.it (V.C.); mariachia.guarino@studenti.unina.it (M.C.G.); pierluigi.giampaolino@unina.it (P.G.); giuseppe.bifulco@unina.it (G.B.); 3 Department of Obstetrics and Gynecology, Villa Sofia Cervello Hospital, University of Palermo, 90146 Palermo, Italy; gaspare.cucinella@unipa.it (G.C.); alessandralopez91@gmail.com (A.L.); 4 Department of Anatomical, Histological, Forensic and Orthopedic Sciences, Departmental Section of Legal Medicine, “Sapienza” University of Rome, 00161 Rome, Italy; simona.zaami@uniroma1.it; 5 Department of Woman, Child and General and Specialized Surgery, University of Campania “Luigi Vanvitelli”, 80138 Naples, Italy; gaetano.riemma@unicampania.it

**Keywords:** vulvar squamous cell carcinoma, inguinofemoral lymph nodes, vulvoscopy, ultrasound, PET, MRI

## Abstract

**Simple Summary:**

Vulvar cancer is a rare gynecological malignant neoplasm that makes necessary accurate preoperative management in order to stage the patient as best as possible, both locally and at the inguinofemoral lymph node level. The aim of this review is to perform a wide evaluation of all the tools that the clinician has at their disposal for a proper diagnosis, such as vulvoscopy, MRI, PET and ultrasound. Vulvoscopy remains essential to carrying out histological diagnosis. Furthermore, for the evaluation of the local extension of the disease and inguinofemoral and/or pelvic lymph nodes, MRI, PET and, more recently, ultrasound are fundamental.

**Abstract:**

Vulvar carcinoma is a rare cancer affecting the genital tract, constituting 4% of gynecological tumors. Vulvar squamous cell carcinoma (VSCC) is the most common type. Diagnosis relies on biopsy during vulvoscopy, plus imaging such as ultrasonography (USG), magnetic resonance imaging (MRI) and positron emission tomography (PET). This review aims to lay out a thorough overview as to the current preoperative management of VSCC, both in case of vulvar and lymph node involvement. The data research was conducted using the following databases: MEDLINE, EMBASE, Web of Sciences, Scopus, ClinicalTrial.gov, OVID and Cochrane Library from 2010 to 2024. The selection criteria included only original articles. Seventeen studies were assessed for eligibility. A concordance rate of 62.3% for vHSIL and 65.2% for carcinoma at vulvoscopy, with a sensitivity of 98%, specificity of 40%, PPV (Positive Predictive Value) of 37% and NPV (Negative Predictive Value) of 98% in identifying malignant lesions was found. Regarding the reliability of PET for staging and assessing lymph node involvement, a mean SUV (Standardized Uptake Value) for malignant vulvar lesions of 8.4 (range 2.5–14.7) was reported. In the case of MRI, useful for the evaluation of loco-regional infiltration and lymph node involvement, the ratio of the short-to-long-axis diameter and the reader’s diagnostic confidence for the presence of lymph node metastasis yielded accuracy of 84.8% and 86.9%, sensitivity of 86.7% and 87.5%, specificity of 81.3% and 86.2%, PPV of 89.7% and 87.5% and NPV of 76.5% and 86.2%, respectively. A long lymph node axis >10 mm and a short diameter >5.8 mm were found to be predictors of malignancy. At USG, instead, the two main characteristics of potentially malignant lymph nodes are cortical thickness and short axis length; the combination of these ultrasound parameters yielded the highest accuracy in distinguishing between negative and positive lymph nodes. Despite the heterogeneity of the included studies and the lack of randomized clinical trials, this review provides a broad overview of the three imaging tools used for the presurgical management of VSCC. Nowadays, although MRI and PET represent the gold standard, ultrasound evaluation is taking on a growing role, as long as it is carried out by expert sonographer. The management of this rare disease should be always performed by a multidisciplinary team in order to precisely stage the tumor and determine the most suitable treatment approach.

## 1. Introduction

Vulvar cancer (VC) is a rare malignant neoplasm of the female genital tract. It represents 4% of gynecological cancers and 0.6% of all tumors in females. Indeed, the percentage of all the new cancer cases defined as VC was 0.3% in 2023, with an equivalent percentage of all cancer deaths [1].

Generally, VC affects postmenopausal women, with an average age at diagnosis greater than 65 years. Different types of VC have been identified, the most frequent being squamous cell carcinoma (SCC), which represents 90% of vulvar neoplasms [2,3].

The rarest histotypes are malignant melanoma, the second most common type of VC, which especially involves the clitoris and the labia minora; extramammary Paget’s disease; adenocarcinoma of the Bartholin’s gland; sarcoma, which unlike other types of VC affects women of all ages, including girls; verrucous carcinoma and basal cell carcinoma [4].

The clinical presentation of VC can be widely varied. The majority of lesions are localized in the labia majora as a single mass or ulcer, but other possible locations may include the labia minora, clitoris, perineum or mons. Furthermore, although many cases may be asymptomatic, itching and burning/pain are quite common [5].

Diagnosis is generally made by biopsy of all suspicious lesions during vulvoscopy, followed by histological examination. The workup presumes personal anamnesis; local and general examination inclusive of evaluation of inguinal lymph nodes, as well as the vulva, vagina and cervix; laboratory tests, such as squamous cell carcinoma (SCC) antigen assay and imaging (positron emission tomography (PET), magnetic resonance imaging (MRI) and ultrasonography (USG)). It is also possible to perform cystoscopy or proctoscopy when deemed necessary. The standard treatment for VC generally includes wide excision or radical vulvectomy, based on size and location, with lymph node staging performed through a minimally invasive surgical technique, such as sentinel lymph node biopsy or inguinofemoral lymphadenectomy [6,7,8].

In this review, we discuss the current state of clinical management of patients with vulvar squamous cell carcinoma (VSCC) in order to clarify the proper preoperative steps and, consequently, to define the best therapeutic approach. Although there are several studies addressing the diagnostics of VC, in our review, in addition to confirming the utility of techniques such as MRI and PET, we focused on the importance of vulvoscopy as an initial approach and especially on the significance of ultrasound, about which there is still much to be understood. The latter, when performed by experienced sonographers, can truly make a difference in the diagnostic process, anticipating a diagnosis of lymph node involvement and allowing for initial guidance on the surgical or therapeutic treatment to be performed. Currently, an increasing number of experts in vulvar carcinoma agree on using ultrasound as a first-line examination for lymph node evaluation, based on specific criteria.

## 2. Materials and Methods

Data research was carried out using the following databases: MEDLINE, EMBASE, Web of Sciences, Scopus, ClinicalTrial.gov, OVID and Cochrane Library, querying for all articles related to vulvar cancer from 2010 up to January 2024.

The studies were identified with the use of a series of the following text words: vulvar carcinoma, vulvar squamous cell cancer, inguinofemoral lymph nodes, groin, ultrasound, PET, MRI and vulvoscopy.

The selection criteria of this review comprised randomized clinical trials, nonrandomized controlled studies (retrospective and observational prospective studies, case-control studies, case series) and review articles.

Conference papers and studies with information coinciding with other publications were not included. In case of overlapping studies, we chose the most recent and/or most inclusive manuscript.

**Figure 1:**
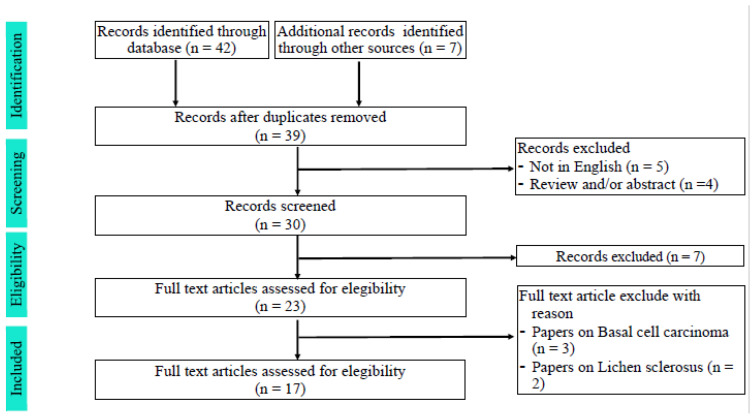
Flowchart of the selection procedure for our review.

We initially selected 42 studies from different databases and 7 studies from additional records; of these, only 30 records were screened. Of these records, 17 studies were assessed for eligibility, whereas 5 were excluded because they were related to preoperative management of basal cell carcinoma and lichen sclerosus (Figure 1).

## 3. Results

From the bibliographic search, a total of seventeen articles were retrieved, of which ten were retrospective [9,10,11,12,13,14,15,16,17,18] and seven were prospective [19,20,21,22,23,24,25].

The objective of this review was to evaluate the reliability of various diagnostic methods commonly used to detect vulvar squamous cell carcinoma (VSCC) and lymph node involvement, in order to carry out an optimal preoperative staging and define the best resulting therapeutic approach.

Although all included studies involved cases of different vulvar tumor types, including melanoma or extramammary Paget’s disease [8], we specifically focused on cases of VSCC, except for vulvar Low-Grade Squamous Intraepithelial Lesion (vLSIL) and vulvar High-Grade Squamous Intraepithelial Lesion (vHSIL) evaluated in vulvoscopy. According to the latest 2015 guidelines from the International Society for the Study of Vulvovaginal Disease (ISSVD), Vulvar Squamous Intraepithelial Lesions are categorized into three types, including the previously mentioned vLSIL and vHSIL, as well as differentiated Vulvar Intraepithelial Neoplasia (dVIN), which is a non-Human-Papilloma-Virus (HPV)-associated intraepithelial neoplasia [7,9] representing a preinvasive lesion of high risk, with a substantial cancer risk of 43.2% [10]. Indeed, patients affected by dVIN are high-risk, where even clinically healthy tissues may exhibit molecular alterations. Despite the complete surgical removal of dVIN lesions, the risk of cancer remains [7,10].

Regarding vulvoscopic examination, our review included two studies specifically focusing on lesions related to HPV [10,11] (see Table 1).

Stuebs et al. focused on the reliability of vulvoscopy by comparing clinical findings with histological results obtained from biopsy [10]. Out of a total of 420 patients, 61 had a diagnosis of vHSIL, while 23 had carcinoma at the final histological examination. Authors evaluated the percentage of patients who had the same result at colposcopy and after biopsy. The results showed a concordance rate of 62.3% for vHSIL and 65.2% for carcinoma [10].

Santoso et al. described, out of a total of 344 patients, the macroscopic characteristics of vLSIL and vHSIL (125/344), dividing them in acetowhite lesions and normal colposcopies. Based on their results, vulvoscopy had a sensitivity of 98%, a specificity of 40%, a positive predictive value (PPV) of 37% and a negative predictive value (NPV) of 98% in identifying malignant lesions [11]. Nine studies regarding the use of PET for staging and assessing lymph node involvement in vulvar carcinoma were included. PET is a recognized and widespread tool for the examination of vulvar tissue and locoregional and distant lymph nodes [12,13,14,15,16,19,20,21,22]. The characteristics of studies on PET are better described in Table 2.

In seven out of the nine studies [12,13,14,16,19,20,21], it was possible to identify the number of patients affected by VSCC and, in the majority of them, to divide patients based on clinical presentation, International Federation of Gynaecology and Obstetrics (FIGO) stage and the size of the primary lesion. All patients who tested positive on PET underwent a specific type of surgery depending on the extension of the disease. The Kamran et al. study is the only one that identifies a mean value related to the standardized uptake value (SUV) of malignant vulvar lesions, with a median of 8.4 (range 2.5–14.7) [19]. On the other hand, different average SUVs of lymph nodes were reported in several studies, and were in all cases greater than two [14,15,16,19,20,21,22] (except for one [22]).

All patients who showed lymph node uptake on PET, unless considered inoperable, underwent ipsilateral or bilateral inguinofemoral lymphadenectomy. According to the values of sensitivity > 50%, specificity > 75%, PPV > 56% and NPV > 57% reported in the included studies, PET appears to be a fundamental imaging tool for the staging of VSCC (Table 2).

As shown in Table 3, the studies evaluating the role of MRI in the management of VSCC focused on the malignancy or benignancy of inguinofemoral lymph nodes.

According to Kataoka et al., the ratio of the short-to-long-axis diameter and the reader’s diagnostic confidence for the presence of lymph node metastasis yielded accuracy of 84.8% and 86.9%, sensitivity of 86.7% and 87.5%, specificity of 81.3% and 86.2%, PPV of 89.7% and 87.5% and NPV of 76.5% and 86.2%, respectively, in groin-by-groin analysis for the prediction of groin lymph node metastasis [17].

Chieko Sakae et al. showed that a long lymph node axis >10 mm and a short diameter > 5.8 mm are predictive of malignancy [18]. The threshold of >10 mm for the long axis gave a sensitivity, specificity, PPV and NPV of 87.5%, 70.6%, 58.3% and 92.3%, respectively, while the threshold of >5.8 mm for the short diameter gave, for the same parameters, the following results: 87.5%, 56%, 41.2% and 87.5%, respectively [18]. As was also demonstrated by Lin et al., MRI shows values greater than 92% for all parameters, including accuracy, in identifying lymph node malignancy [20].

Although it is a recent approach and depends on the operator’s skills, ultrasonography, which can represent a first-line examination due to its speed of execution and the lack of need for a contrast medium, is considered of primary importance in the evaluation of inguinal lymph nodes in patients affected by VSCC.

Table 4 includes all studies in which ultrasound features of potentially malignant lymph nodes are evaluated, including cortical thickness, short axis, cortical interruption, vascular flow localization, cortical–medullar interface distortion, vascular flow architecture pattern, grouping and cortical thickening.

Garganese et al. identified the two main characteristics of potentially malignant lymph nodes: cortical thickness and short axis length of the dominant lymph node (LN) [23].

The parameters mentioned above demonstrated the highest accuracy, striking a fine balance between specificity and sensitivity in predicting negative LN status: the dominant LN’s cortical thickness with a threshold at 2.5 mm had sensitivity at 90.0%, specificity at 77.9%, PPV at 58.7%, NPV at 95.7%, accuracy at 81% and the short axis length of the dominant LN with a threshold at 8.4 mm had sensitivity at 63.9%, specificity at 90.6%, PPV at 74.2%, NPV at 85.6%, accuracy at 82.6% [23].

The study carried out by Power et al. underscored the crucial role of regular follow-up by the use of groin ultrasound in the timely detection of groin metastasis. Their findings showed that ultrasound sensitivity in identifying groin metastasis was 100% (95% CI 16–100%), with a specificity of 92% (95% CI 89–95%) [24].

According to the Morphnode study conducted by Fragomeni et al., among ultrasound variables, cortical thickness and short axis diameter had the highest NPV (85.0%), and the sensitivity and specificity values of these two variables were 72.3% and 73.4%, 71.8% and 73.3%, respectively. The similar results obtained in the studies reported in Table 4 demonstrate that ultrasound is a minimally invasive and highly recommended examination in the evaluation of potentially malignant inguinofemoral lymph nodes [24,25,26].

## 4. Discussion

As mentioned earlier, this review includes data from a total of 17 original studies, none of them randomized, wherein the main diagnostic elements of each of the three techniques, ultrasound, MRI and PET, for VSCC have been analyzed.

A good imaging evaluation must be functional for the subsequent surgical approach. International guidelines, such as the recent National Comprehensive Cancer Network guidelines, recommend the use of several diagnostic tools, including vulvoscopy focusing on macroscopic aspects of the primary lesion, MRI to identify the local extent of the disease and lymph node involvement, PET to assess tumor SUV levels and any uptake at the lymph node level [27,28] and, ultimately, nodal ultrasound assessment which, thanks to recently published studies, represents a fundamentally non-invasive and economically accessible investigation into the characterization of metastatic lymph nodes [23,24,25,26].

The first approach to vulvar cancer is represented by the clinical examination. Despite the limited number of published studies on the matter, it has been established that through vulvoscopy it is possible, first of all, to assess whether the lesion is potentially benign or malignant and to perform targeted biopsies before moving on to the subsequent diagnostic steps [10,11,29].

Stuebs et al. demonstrated how vulvoscopy enables the physician to evaluate vulvar lesions with greater precision compared to those carried out without a colposcope. They showed how the integration of both methods, namely naked-eye examination first and then the use of the colposcope, can provide a comprehensive and accurate analysis of the lesion, allowing for the identification of areas for biopsy, as well as showing the margins and extent of the lesion. In this study, the concordance between the clinical observations and the ultimate histology results from punch biopsies was evaluated [10]. Thanks to the development of a new classification aimed at simplifying the categorization of vulvoscopic findings, Stuebs et al. showed that the concordance rate between clinical findings and histological evaluation of biopsy samples is greater than 50% in cases of suspected vHSIL and carcinomas. It is also well known that the use of acetic acid is important in recognizing potentially malignant lesions [10,11].

In this regard, Santoso et al. precisely evaluated vulvar acetowhite lesions as predictors for vLSIL and vHSIL [11]. Out of the 241 patients with acetowhite lesions, 89 were confirmed to have true high-grade dysplasia, showing that acetowhitening of the vulva has high sensitivity as a predictor of vHSIL [11].

PET is a functional imaging technique that utilizes a radiotracer, typically a radioactive glucose analog called F18 fluorodeoxyglucose (FDG), to identify malignant tumors which consume more glucose than benign tissue, a phenomenon known as the Warburg effect. All published studies to date demonstrate the consistent utility of PET in detecting lymph nodes and distant metastases from vulvar carcinoma [12,13,14,15,16,19,20,21,22,30,31].

In our review, we attempted to group a significant number of studies published over a period of more than 10 years to understand if there could be a standard SUV value associated with lymph node metastases from vulvar carcinoma. Data from literature revealed different criteria used to evaluate nodal disease, which could explain variations in their detection. For these reasons, given the lack of uniform criteria, further evaluations are necessary.

The study by Oldan et al., given the uncertainties associated with the evaluation of SUV max, is the only study among those cited that employs FDG PET-CT with both quantitative and non-quantitative approaches for lymph node assessment. They demonstrated how, using a semiquantitative approach that compares the nodal SUV with an SUV > 2, the mean standardized uptake value of the liver could be utilized as a standardized criterion for a proper interpretation. Using an SUV max cut off of 4.5, two times the average liver uptake, they obtained a 100% sensitivity and 89% specificity for positive inguinal nodes [15]. Dolanbay et al. demonstrated the difference between metastatic and reactive lymph nodes on PET, with the minimum SUV of a metastatic node being 3.5, while the highest SUV max value for a reactive node was 3.1 [21].

Regarding MRI, the international guidelines recommend it as the preferred tool for assessing vulvar tissue due to its resolution power, in particular for tumors larger than 2 cm that extend beyond the vulva and perineum and those with stromal invasion greater than 1 mm [32]. In comparison to muscles, tumors typically appear as solid and hypo- to iso-intense on T1-weighted imaging, and as intermediate to hyper-intense (“evil gray”) masses on T2-weighted imaging sequences [32].

According to a recent review by Chow et al., MRI imaging offers several advantages. It provides superior contrast resolution and precise delineation of soft tissues, making it the most sensitive modality for detecting lymph node involvement. Moreover, it is preferred for assessing local invasion and treatment response [33].

Lymph node involvement can be highlighted on MRI through various criteria, such as a short axis diameter exceeding 1 cm, a rounded shape with a long axis to short axis ratio less than 1.3:1, the presence of cystic changes within the node, absence of fatty hilum and irregular shape [32]. We have focused on identifying the characteristics of metastatic lymph nodes on MRI.

The study by Kataoka et al. showed that MRI is highly accurate in determining tumor size, characteristics and dimensions of lymph node metastases [17]. They focused especially on the data regarding the reader’s diagnostic confidence for the presence of lymph node metastases, which showed the highest sensitivity (93%) and accuracy (87%), as well as the length-to-width ratio (S/L ratio) of ≥0.75 for diagnosing lymph node metastases [17].

The prospective study by Lin et al. investigating the effectiveness of PET and MRI in the management of vulvar carcinoma demonstrated that, while in primary staging, MRI was more effective than PET in recognizing pelvic lymph nodes or distant metastases, and there was no difference in recognizing metastases at the level of inguinal lymph nodes [20]. The advantage of MRI over PET is that it recognizes the anatomical characteristics of lymph nodes, which PET is unable to do.

Thanks to recent studies by Garganese et al., Fragomeni et al. and Fisherova et al. [23,25,34], the role of ultrasound has become increasingly relevant in the management of vulvar neoplasia. Even though MRI and PET are recommended by international guidelines [27], ultrasound alone could be sufficient for lymph node staging in vulvar cancer and for preoperative assessment, if performed by trained sonographers in centers with access to high-end ultrasound machines and expert sonologists [32].

The consensus opinion from the Vulvar International Tumor Analysis (VITA) group should help to solve the issue related to the lack of standardized ultrasound nomenclature to describe lymph nodes regarding terms, definitions and measurements.

Indeed, the aim of this consensus was to introduce a standardized method for describing the ultrasound characteristics of inguinal lymph nodes in patients with vulvar carcinoma, which may be used to describe inguinal lymph nodes on grayscale and color/power Doppler ultrasound [34].

When performed by skilled sonographers, ultrasound has shown high values of sensitivity, specificity, PPV, NPV and diagnostic accuracy in detecting positive lymph nodes in patients with demonstrating vulvar cancer [23,24,25,26].

Garganese et al., in their MorphoNode study, reported on how the combination of two ultrasound parameters (S length and C/M thickness ratio) yielded the highest accuracy in distinguishing between negative and positive lymph nodes (sensitivity, 88.9%; specificity, 82.4%) [23].

Even Fragomeni et al. demonstrated how ultrasound is an accurate method in the preoperative evaluation of inguinofemoral lymph nodes, aiming to create a multi-modular model based on machine learning to discriminate between metastatic and non-metastatic inguinal lymph nodes in patients with vulvar cancer.

They utilized fourteen informative features to train and test the machine in order to obtain a diagnostic model that could be easily integrated into clinical practice for preoperative stratification of vulvar cancer patients. The Morphonode Predictive Model showed how a specific data classifier, known as a random forest classifier, predicted metastatic/non-metastatic lymph nodes with an accuracy of 93.3% and a NPV of 97.1% [25].

## 5. Conclusions

In this review, we aimed to provide an updated overview of the management of VSCC, which, although rare, has a high impact on patients’ lives.

Therefore, it is important to know the main diagnostic steps that allow for the staging of this neoplasm and the appropriate therapeutic approach. Despite the important heterogeneity of the included studies, the strength of our study lies in the fact that it represents the most complete review on this topic, highlighting the importance of integrating various techniques such as vulvoscopy, MRI, PET and ultrasound to achieve the most accurate diagnosis through the evaluation of several aspects of VSCC, in order to plan the best treatment for patients.

Although guidelines recommend the use of MRI and PET in staging VSCC, ultrasound is gaining increasing relevance, as demonstrated by recent studies aimed at identifying patterns based on unique assessment criteria.

We think that ultrasound, which represents the least invasive and most accessible option for patients thanks to the training of increasingly qualified sonographers and improvements in technology, can become equivalent to MRI or PET in the evaluation of lymph node metastasis, just as it is becoming so for ovarian cancer in qualified gynecological ultrasound centers [33,34,35,36,37].

## Figures and Tables

**Table 1:** Vulvoscopy: the first vulvar cancer diagnostic approach.

**Author, Year**	Stuebs et al., 2020 [9]	Santoso et al., 2015 [10]
**Country**	Germany	Germany
**Type of Study**	Retrospective	Retrospective
**Number of Patients**	420	344
**Age**	47.6 ± 17 ^a^	34 (19–65) ^b^
**Macroscopic Features**	Nr	vSIL: hyperpigmentation, ulceration, thickening
**Histological Findings on Punch Biopsy ^c^**	vHSIL: 61/420 (14.5%) Carcinoma: 23/420 (5.5%)	Nr
**Clinical Findings**	38/61 (62.3%) ^d^ 15/23 (65.2%)	Acetowhite colposcopy ^e^ vLSIL: 32/125 (25.6%) vHSIL: 88/125 (70.4%) Normal colposcopy vLSIL: 3/125 (2.4%) vHSIL: 2/125 (1.6%)
**Concordance Rate of Clinical Findings ^f^**	vHSIL: 62.3% Carcinoma: 65.2%	Nr
**Sensitivity**	Nr	98%
**Specificity**	Nr	40%
**PPV**	Nr	37%
**NPV**	Nr	98%

^a^ Mean age ± standard deviation; ^b^ median (range); ^c^ total number of vulvar high-grade squamous intraepithelial lesions (vHSIL) and carcinomas confirmed upon histological examination; ^d^ number of clinical findings out of the total corresponding histological findings for vHSIL and carcinoma; ^e^ number of acetowhite and normal colposcopies out of the total cases of vulvar intraepithelial lesions; ^f^ percentage of patients with the same clinical findings at the clinical examination and biopsy; Nr: not reported.

**Table 2:** PET: functional examination of vulvar, lymph node and distant disease.

Author, Year	Peiró et al., 2014 [11]	Kamran et al., 2014 [12]	Lin et al., 2014 [19]	Dolanbay et al., 2015 [20]	Collarino et al., 2017 [21]	Garganese et al., 2017 [22]	Crivellaro et al., 2017 [13]	Oldan et al., 2018 [14]	Rufini et al., 2021 [15]
**Country**	Spain	Ireland	Taiwan	Germany	Netherlands	Italy	Italy	United States	Italy
**Type of Study**	Retrospective	Retrospective	Prospective	Prospective	Prospective	Prospective	Retrospective	Retrospective	Retrospective
**Number of Patients**	10	20	23	8	33	47	29	96	160
**Age**	64.5 (30–81) ^b^	59 (38–83) ^b^	64.7 ± 16.2 ^a^	64.50 ± 13.25 ^a^	69 ± 13.4 ^a^	71 (38–87) ^b^	69 (51–88) ^b^	55 ± 11 ^a^	70.6 ± 12.6 ^a^
**Histological Type: Squamous**	4/10 (40%) ^c^	20 (100%)	15/23 (65.2%)	8 (100%)	Nr	45 (95.7%)	9 (31%)	Nr	143 (89.4%)
**Clinical Presentation**									
cN0	3/4 (75%)	8 (40%)	8/12 (66.7%) ^e^	0	13 (39.3%)	Nr	15 (51.7%)	Nr	96 (60%)
cN+	1/4 (25%)	12 (60%)	4/12 (33.3%)	8 (100%)	20 (60.7%)	Nr	14 (48.3%)	15/21 (71.4%) ^h^	64 (40%)
**Figo Stage**									
I II III IV IVA IVB	Nr	8 (I/II) (40%) 12 (III) (60%)	6/12 (50%) 2/12 (16.6%) 4/12 (43.4%	4 (50%) 4(50%)	15 (45.5%) 3 (9%) 15 (45.5%)	18 (38%) 13 (28%) 13 (28%)	2 (7%) 13 (45%) 12 (41%) 2 (7%)	Nr	86 (53.8%) 4 (2.5%) 57 (35.6%) 13 (8.1%) 2 (1.2%) 11 (6.8%)
**Focality**									
Unifocal	3/4 (75%)	11 (55%)	Nr	Nr	27 (82%)	12 (26%) ^g^	Nr	Nr	142/157 (90.4%) ^i^
Multifocal	1/4 (25%)	9 (45%)	Nr	Nr	6 (18%)	9 (19%) ^g^	Nr	Nr	15/157 (9.6%) ^i^
**Size of Primary Lesion**						28 (7–75) ^b^		1.95 (0.3–8.6) ^b^	
≤40mm	Nr	Nr	7/12(58.3%)	5 (62.5%)	25 (76%)	Nr	Nr	Nr	102/155 (65.8%) ^j^
≥40 mm	Nr	Nr	5/12 (41.7%)	3 (37.5%)	8 (24%)	Nr	Nr	Nr	53 (34.2%) ^j^
**Grading**									
G1 G2 G3	Nr	4 (20%) 12 (60%) 4 (20%)	Nr Nr Nr	Nr	4 (12%) 24 (73%) 5 (15%)	4 (9%) 34 (74%) 8 (17%)	Nr	Nr	12/154 (7.8%) 105/154 (68%) 27/154 (6.5%) 10/154 (6.5%) ^k^
**Treatment ^d^**	1/4 (25%) HV + UGND 1/4 (25%) RV + UGND 1/4 (25%) QT + RV + PH 1/4 (25%) RV + BGND	15 (75%) RVE + BGND 2 (10%) RVE + UGND 3 (15%) MS	1/12 (8.3%) RV + BGND + PGND + RT 1/12(8.3%) RV + BGND + RT 2/12 (16.6%) RVE + BGND 2/12 (16.6%) RV + BGND 1/12 (8.3%) RVE + BGND + CCRT 1/12 (8.3%) RV + BGND + PGND + RT 1/12 (8.3%) RV + BGND + CCRT 1/12 (8.3%) RVE + BGND 1/12 (8.3%) RV + UGND + RT 1/12 (8.3%) RV + BGND	7/8 (87.5%) RVE + UGND 1/8 (12.5%) RVE + BGND	11 (33%) PV 22 (67%) RV 9 (27%) UGND 24 (73%) BGND	16 (34%) PV 31 (66%) RV 21 (44%) UGND 26 (55%) BGND	23 (79.3%) BGND 6 (20.7%) UGND	15/21 (71.4%) ^h^ BGND	44 (27.5%) PV 89 (55.6%) RV 24 (15.0%) UGND 136 (85.0%) BGND
**SUV Max and** **Range**						6.1 (0.7–16.2)			
Vulvar	Nr	8.4 (2.5–14.7) ^b^	Nr	Nr	Nr	Nr	Nr	Nr	Nr
Groin	Nr	2.1 (2.4–3) ^b^	>3.0	3.5-15 ^f^	>1.32	Nr	6.1 (0.7–16.2)	>4.5	2.03
**Sensitivity**	Nr	50%	92%	100%	95.2%	56%	53%	100%	75%
**Specificity**	Nr	100%	91%	100%	75%	88%	85%	89%	76.7%
**PPV**	95%	100%	85%	100%	69%	38%	76%	Nr	56.3%
**NPV**	Nr	57.1%	95%	100%	96.4%	93%	67%	Nr	88.5%

^a^ Mean ± standard deviation; ^b^ median (range); ^c^ 4/10 tumors are squamous cell carcinoma; ^d^ RVE: radical vulvar excision (2 cm horizontal margin beyond the tumor and excision down to the deep fascia or periosteum); RV: radical vulvectomy; PV: partial vulvectomy; HV: hemivulvectomy; UGND: unilateral groin node dissection; BGND: bilateral groin node dissection; PH: posterior hemivulvectomy; MS: major surgery; PGND: pelvic groin node dissection; QT: chemotherapy; RT: radiotherapy; ^e^ scans were performed on 12 patients with untreated squamous vulvar carcinoma; ^f^ SUV value was identified in four metastatic groins (50%). It is impossible to identify a clear cut-off value due to the small sample size in this study. These data may help in the identification of a cut-off value if compared with a larger series. ^g^ The remaining patients were those who had a primary lesion completely removed during prior diagnostic excisional surgery, patients with evidence of unilateral and contralateral groin node involvement and those with a disease relapse after prior surgical and/or medical treatments. ^h^ The total number of patients who underwent inguinal lymph node evaluation is 21. ^i^ Data available for 157 patients; ^j^ information available for 155 patients; ^k^ information available for 154 patients; Na: not applicable; Nr: not reported.

**Table 3:** MRI: the tool to evaluate the extent of the local disease and its characteristics.

**Author, Year**	Kataoka et al., 2010 [16]	Lin et al., 2014 [19]	Sakae et al., 2016 [17]
**Country**	United Kingdom	Taiwan	Japan
**Type of Study**	Retrospective	Prospective	Retrospective
**Vulvar Features**	Fat suppression on T2-weighted images	Nr	Nr
**Groin Features**	- Short-axis diameter more than 5 mm - Contour (1, smooth; 2, lobular; 3, irregular; 4, spiculated) - Presence of cystic changes/necrosis, loss of fatty hilum - Similarity of signal intensity to the primary lesion - Reader’s diagnostic confidence for presence of metastasis	Nr	**- Positive groins:** Mean long axis: 12.8 (>10 mm) Short diameter mean: 9.2 (>5.8 mm) S/L ratio: 0.73 **- Negative groins:** Mean long axis: 8.8 mm (≤10 mm) Short diameter mean: 6.7 (≤5.8 mm) S/L ratio: 0.77
**Number of Patients**	49	23	41
**Age**	70.4 (40–88) ^b^	64.7 ± 16.2 ^a^	71 (28–91) ^b^
**Histological Type: Squamous**	Nr	15/23 (65.2%)	32 (78%)
**Clinical Presentation** CN0 CN+	Nr Nr	8/12 (66.7%) ^e^ 4/12 (33.3%) ^e^	13/38 ^f^ (34.2%) 25/38 ^f^ (65.8%)
**FIGO Stage**			
I II III IV IVA IVB	7/36 (19.4%) ^c^ 9/36 (25%) ^c^ 11/36 (30.5%) ^c^ 8/36 (22.2%) ^c^	6/12 (50%) ^e^ 2/12 (16.6%) ^e^ 4/12 (43.4%) ^e^	23 (56%) 3 (7.3%) 10 (24.4%) 5 (12.2%)
**Size of Primary Lesion**			
≤40 mm ≥40 mm	<2 cm 12 (24.5%) >2 cm 37/49 (75.5%) Nr	7/12 (58.3%) ^e^ 5/12 (41.7%) ^e^	Nr Nr
			Nr
**Treatment ^d^**	8 (16.32%) RVE 8 (16.3%) RVE + UGND 8 (16.3%) RVE + BGND 8 (16.3%) RVE + SGND 2/49 (4%) RV 1 (2%) RV + UGND 8 (16.3%) RV + BGND 0 (0%) PH 1 (2%) PH + UGND 4 (4%) PH + BGND	1/12 (8.3%) ^e^ RV + BGND + PGND + RT 1/12 (8.3%) RV + BGND + RT 2/12 (16.6%) RVE + BGND 2/12 (16.6%) RV + BGND 1/12 (8.3%) RVE + BGND + CCRT 1/12 (8.3%) RV + BGND + PGND + RT 1/12 (8.3%) RV + BGND + CCRT 1/12 (8.3%) RVE + BGND 1/12 (8.3%) RV + UGND + RT 1/12 (8.3%) RV + BGND	Nr
**Sensitivity**	- S/L ratio ≥ 0.75 86.7% - Readers’ confidence of metastasis 87.5%	92%	- Long axis > 10.0 mm 87.5% - Short axis > 5.8 mm 87.5%
**Specificity**	- S/L ratio ≥ 0.75 81.3% - Readers’ confidence of metastasis 86.2%	100%	- Long axis > 10.0 mm 70.6% - Short axis > 5.8 mm 41.2%
**PPV**	- S/L ratio ≥ 0.75 89.7% - Readers’ confidence of metastasis 87.5%	100%	- Long axis > 10.0 mm 58.3% - Short axis > 5.8 mm 56.0%
**NPV**	- S/L ratio ≥ 0.75 76.5% - Readers’ confidence of metastasis 86.2%	96%	- Long axis > 10.0 mm 92.3% - Short axis > 5.8 mm 41.2%
**Accuracy**	- S/L ratio ≥ 0.75 84.8% - Readers’ confidence of metastasis 86.9%	97%	- Long axis > 10.0 mm 76.0% - Short axis > 5.8 mm 87.5%

^a^ Mean ± standard deviation; ^b^ median (range); ^c^ 36 patients with primary cancer; ^d^ RVE: radical vulvar excision (2 cm horizontal margin beyond the tumor and excision down to the deep fascia or periosteum); RV: radical vulvectomy; PV: partial vulvectomy; HV: hemivulvectomy; UGND: unilateral groin node dissection; BGND: bilateral groin node dissection; PH: posterior hemivulvectomy; MS: major surgery; PGND: pelvic groin node dissection; QT: chemotherapy; RT: radiotherapy; ^e^ PET scans were performed on 12 patients with untreated squamous vulvar carcinoma; ^f^ preoperative MRI scans were performed on 38 patients; Nr: not reported.

**Table 4:** Ultrasound: the tool for the evaluation of suspected inguino-femoral lymph nodes.

**Author, Year**	Pouwer et al., 2018 [23]	Garganese et al., 2020 [18]	Fragomeni et al., 2023 [24]	Hacker et al., 2023 [25]
**Country**	Netherlands	Italy	Italy	Australia
**Type of Study**	Prospective	Retrospective observational	Prospective	Prospective pilot
**Number of Patients**	76	144	127	32
**Age**	67 (37–89) ^a^	74 (16–94) ^a^	69 (32–95) ^a^	64.5 (35–88) ^a^
**Clinical Presentation**				
cN0	76 (100%)	87/144 (60.4%)	71(56%)	26 (81.25%)
cN+		57/144 (39.6%)	56 (44%)	6 (18.75%)
**Focality**				
Unifocal	76 (100%)	133/143 (93%) ^b^	106 (83.5%)	Nr
Multifocal		10/143 (7%) ^b^	21 (16.5%)	Nr
**Histotype**				
Squamous	76 (100%)	132/144 (91.7%)	110 (86.6%)	Nr
**Size of Primary Lesion**				10 (6–20) ^a^
<20 mm		39/141 (27.7%) ^c^	28/122 (23%) ^f^	Nr
20–40 mm	76 (<40 mm)	53/141 (37.6%) ^c^	52/122 (42.6%) ^f^	Nr
>40 mm		49/141 (34.8%) ^c^	42/122 (24.4%) ^f^	Nr
**Stage**				
I	76 (100%)	75/139 (54%) ^d^	59 (46.5%)	Nr
II		8/139 (5.8%) ^d^	4 (3.2%)	Nr
III		47/139 (33.8%) ^d^	40 (31.5%)	Nr
IV		9/139 (6.5%) ^d^	4 (3.2%)	Nr
**Grading**				
G1	25 (33%)	23/133 (17.3%) ^e^	20/110 (18.18%) ^g^	Nr
G2	43 (57%)	92/133 (69.2%) ^e^	62/110 (56.4) ^g^	Nr
G3	8(10%)	18/133 (13.5%) ^e^	19/110 (17.3%) ^g^	Nr
**Ultrasound Variables’ Performances**	- Short-axis diameter ≥ 10 mm in oval-shaped lymph nodes or ≥8 mm in circular lymph nodes - Hilar hypoechogenicity - General attenuation - Irregularity of the margin - Abnormal vascular pattern on Doppler	- Globular shape - Cortical thickening - Hilum anomalies - Inhomogeneous echostructure - Intranodal deposits - Cortical interruption - Perinodal hyperechoic ring - Rich vascularization - Nodal grouping - Cortical thickness of dominant LN (mm): 2.5 mm - Short axes, length of the dominant LN 8.4 mm - C/M Ratio 1.2 mm	- Cortical thickness >2 mm - Short axis >8 mm - Nodal core sign absence - Perinodal hyperecogenic ring - Cortical interruption - Echogenicity inhomogeneous - Focal intranodal deposit present -Vascular flow localization: 2, 3, 4 - Cortical–medullar interface distortion present - Vascular flow architecture pattern: 1-2-3 - Color score 3-4 - Shape asymmetric - Nodal core sign absent - Cortical interruption suspect or present - Grouping moderate or complete - Cortical thickening present 1, 3, 4	- Generalized or focal cortical thickening - Inhomogeneity of the texture of either the cortex or medulla - Absence of the medulla or hilum - Focal masses within the cortex deforming or disrupting the junction with either the medulla or capsule - Evidence of lymph node matting - Evidence of an abnormal vascular pattern on Doppler
**Sensitivity**	100%	- Cortical thickness of the dominant LN 90% - Short axes, length of the dominant LN 63.9%	- Cortical thickness 72.3% - Short axes 73.4%	100%
**Specificity**	92%	- Cortical thickness of the dominant LN 77.9% - Short axes, length of the dominant LN 90.6%	- Cortical thickness 71.8% - Short axes 73.3%	97%
**PPV**	68%	- Cortical thickness of the dominant LN 58.7% - Short axes, length of the dominant LN 74.2%	Nr	75%
**NPV**	100%	- Cortical thickness of the dominant LN 95.7% - Short axes, length of the dominant LN 85.6%	- Cortical thickness 85% - Short axes 85%	100%
**Accuracy**	Nr	- Cortical thickness of the dominant LN 81.0% - Short axes, length of the dominant LN 82.6%	Nr	Nr

^a^ Median (range). Data available for: ^b^ 143 patients; ^c^ 141 patients; ^d^ 139 patients; ^e^ 133 patients; ^f^ 122 patients; ^g^ 110 patients. Nr: Not reported.
